# Apelin Deficiency Accelerates the Progression of Amyotrophic Lateral Sclerosis

**DOI:** 10.1371/journal.pone.0023968

**Published:** 2011-08-24

**Authors:** Atsushi Kasai, Toshihiko Kinjo, Rie Ishihara, Ikumi Sakai, Yuki Ishimaru, Yasuhiro Yoshioka, Akiko Yamamuro, Kumiko Ishige, Yoshihisa Ito, Sadaaki Maeda

**Affiliations:** 1 Department of Pharmacotherapeutics, Faculty of Pharmaceutical Sciences, Setsunan University, Hirakata, Osaka, Japan; 2 Laboratory of Pharmacology, Department of Pharmacy, School of Pharmacy, Nihon University. Funabashi, Chiba, Japan; 3 Department of Psychiatry, Semel Institute for Neuroscience and Human Behavior, University of California Los Angeles, Los Angeles, California, United States of America; Claremont Colleges, United States of America

## Abstract

Amyotrophic lateral sclerosis (ALS) is a neurodegenerative disease characterized by the selective loss of motor neurons. Recent studies have implicated that chronic hypoxia and insufficient vascular endothelial growth factor (VEGF)-dependent neuroprotection may lead to the degeneration of motor neurons in ALS. Expression of apelin, an endogenous ligand for the G protein-coupled receptor APJ, is regulated by hypoxia. In addition, recent reports suggest that apelin protects neurons against glutamate-induced excitotoxicity. Here, we examined whether apelin is an endogenous neuroprotective factor using SOD1^G93A^ mouse model of ALS. In mouse CNS tissues, the highest expressions of both apelin and APJ mRNAs were detected in spinal cord. APJ immunoreactivity was observed in neuronal cell bodies located in gray matter of spinal cord. Although apelin mRNA expression in the spinal cord of wild-type mice was not changed from 4 to 18 weeks age, that of SOD1^G93A^ mice was reduced along with the paralytic phenotype. In addition, double mutant apelin-deficient and SOD1^G93A^ displayed the disease phenotypes earlier than SOD1^G93A^ littermates. Immunohistochemical observation revealed that the number of motor neurons was decreased and microglia were activated in the spinal cord of the double mutant mice, indicating that apelin deficiency pathologically accelerated the progression of ALS. Furthermore, we showed that apelin enhanced the protective effect of VEGF on H_2_O_2_-induced neuronal death in primary neurons. These results suggest that apelin/APJ system in the spinal cord has a neuroprotective effect against the pathogenesis of ALS.

## Introduction

Amyotrophic lateral sclerosis (ALS), the most common adult-onset motor neuron disease, is caused by a selective loss of motor neurons leading to progressive paralysis of muscles and ultimately death [1]. Approximately 10% of ALS cases is familial, of which 20% are associated with dominant mutations in the gene encoding the human Cu^2+^/Zn^2+^ superoxide dismutase-1 (SOD1) [2]. Transgenic animals overexpressing mutant SOD1 develop progressive motor neuron disease that resembles the clinical and pathological features of human familial ALS [3]. Although the precise pathogenesis of ALS remains unclear, studies using these animal models have proposed several hypotheses to explain motor neuron degeneration, including glutamate-induced excitotoxicity [4] and oxidative damage [1].

Recently, chronic hypoxia and insufficient vascular endothelial growth factor (VEGF)-dependent neuroprotection has been linked to the degeneration of motor neurons in ALS. Mice with deletion of the hypoxia-response element in the VEGF promoter region (VEGF^δ/δ^) develop spinal hypoperfusion and adult-onset progressive motor neuron degeneration [5]. Prior to motor neuron degeneration, SOD1 mutants exhibit disruption of the blood-spinal cord barrier, endothelial damage, and hypoperfusion [6]. Furthermore, Crossbreeding SOD1^G93A^ mutant mice with mice overexpressing VEGF in neurons produces a phenotype characterized by delayed motoneuron loss and motor impairment, and prolonged survival compared with mice carrying the SOD1^G93A^ gene alone [7]. In addition, the cerebrospinal fluid VEGF levels are significantly increased in patients with long duration of ALS [8]. These findings suggest that chronic hypoxia have been implicated in the pathology of ALS.

The human *apj* gene encodes a G protein-coupled receptor (GPCR) [9]. Apelin was identified as an endogenous ligand for the orphan GPCR APJ [10]. Apelin expression is high in human spinal cord [11], and regulated by hypoxia [12], [13]. Recent studies suggest that apelin protects neurons against glutamate-induced excitotoxicity [14], [15]. Furthermore, we previously showed that the apelin/APJ system cooperates with VEGF to regulate vascular development in the retina [16]. Kidoya et al. also demonstrated that apelin in combination with VEGF induced proliferation and assembly of endothelial cells [17]. Therefore, the apelin/APJ system alone or in combination with VEGF may play a neuroprotective role in the pathogenesis of ALS. Here, to examine whether apelin is an endogenous neuroprotective factor using the SOD1^G93A^ mouse model of ALS, we have established apelin deficient mice carrying the SOD1 mutation. In the present study, we demonstrated that the apelin receptor APJ was detected in neuronal cell bodies located in spinal cord, that apelin expression in spinal cord of SOD1^G93A^ mice was reduced along with the paralytic phenotype, and that apelin deficiency accelerated the progression of ALS. In addition to *in vivo* studies using transgenic mice, our *in vitro* studies showed that apelin enhanced the protective effect of VEGF on oxidative stress-induced neuronal death. Our findings suggest that apelin/APJ system plays a protective role in the pathogenesis of ALS.

## Results

### Apelin and APJ expressions in mouse CNS tissues

**Figure 1:**
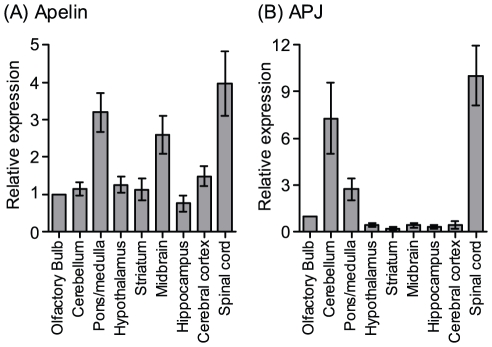
Apelin and APJ mRNA expression profiles in mouse CNS tissues. Expression of apelin (A) and APJ (B) in wild type mice were examined by real-time PCR (n = 3–4). Data are expressed as arbitrary units normalized to β-actin, and represent mean ± SEM.

We analyzed the precise distribution of apelin and APJ mRNA in mouse CNS tissues by real-time RT-PCR method. As shown in Fig. 1, the highest expression of apelin was observed in the spinal cord (4-fold higher than olfactory bulb, Fig. 1A). APJ receptor expression was also highest in the spinal cord (Fig. 1B).

**Figure 2:**
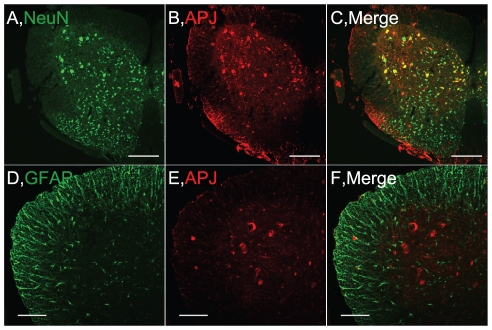
APJ expression in the lumbar spinal cord of WT mice. Representative pictures of ventral horn show double immunostaining for APJ and NeuN (A–C) or GFAP (D–F) in the lumbar spinal cord. Scale bar = 200 µm (upper panels), 100 µm (lower panels).

To identify the target cells for apelin/APJ system in spinal cord, we examined APJ expression in the spinal cord by immunohistochemical staining. In the spinal cord from wild-type mice at 14 weeks of age, APJ-positive cells were located in the ventral horn and around the central canal, which were colocalized with NeuN, a neuronal marker, -positive cells throughout the spinal cord gray matter (Fig. 2A–C). In contrast, APJ was expressed in neither glial fibrillary acidic protein (GFAP)-positive glial cells Fig. 2D–F) nor IbaI-positive microglia (data not shown) in spinal cord. In addition that the APJ-positive cells were in the large neurons of ventral horn, we addressed APJ was expressed in the choline acetyltansferase (ChAT)-positive cells by immunostaining in adjacent sections (data not shown). These data indicated that APJ was expressed in the motor neurons.

### Apelin mRNA expression is downregulated in spinal cords of SOD1^G93A^ mice

**Figure 3:**
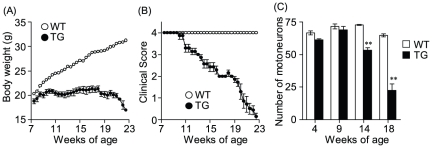
Time course of disease progression in SOD1^G93A^ mice monitored with three different tests. (A) Motor signs (hind limb tremors) were measured with the clinical scoring system (see methods). (B) Body weight was monitored twice a week. (C) The number of ChAT-positive cell bodies was decreased at end-stage disease for SOD1^G93A^ mice. **p<0.01 vs. wild-type mice. Data represent mean ± SEM.

The first clinical signs of motor neuron disease in SOD1^G93A^ mice were fine hind limb tremors. The SOD1^G93A^ mice began to display hind limb tremors around 10 weeks, gait abnormalities around 14 weeks, and dragging of at least one hind limb around 18 weeks, whereas the control mice never showed signs of motor dysfunction (Fig. 3A). Contrasted with the progressive weight gain of the control group, body weight loss in SOD1^G93A^ mice became evident around 10 weeks of age (Fig. 3B). Furthermore, we investigated the number of motor neuron in the lumbar spinal cord sections from 4-week-old to adult mice by ChAT immunostaining. Compared with control group, SOD1^G93A^ mice began to show prominent depletion of anterior horn motor neurons around 14 weeks age (Fig. 3C).

**Figure 4:**
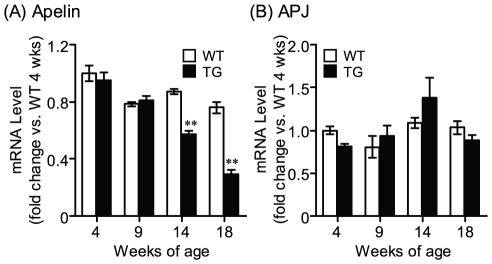
Gene expression in lumbar spinal cord of SOD1^G93A^ mice. Temporal expression patterns of apelin (A) and APJ (B) in the lumbar spinal cord of wild-type (open column) and SOD1^G93A^ (closed column) mice were examined by real-time RT-PCR (n = 3–6). Data are mean ± SEM. **p<0.01 vs. wild-type mice.

We next compared the expressions of apelin and APJ mRNA in lumbar spinal cord of SOD1^G93A^ mice with those of control mice using real-time RT-PCR. In SOD1^G93A^ mice, apelin expression in the spinal cord was significantly decreased at the 14 and 18 weeks age (Fig. 4A). In contrast to spinal cord, apelin expression in the lung of SOD1^G93A^ mice was similar to that of control mice at the 18 weeks age (Control: 1.00±0.11, TG: 1.00±0.12). On the other hand, there was not a significant difference in APJ expression in lumbar spinal cord of between control and SOD1^G93A^ mice (Fig. 4B).

### Apelin deficiency accelerates the progression of the disease in SOD1^G93A^ mice

**Figure 5:**
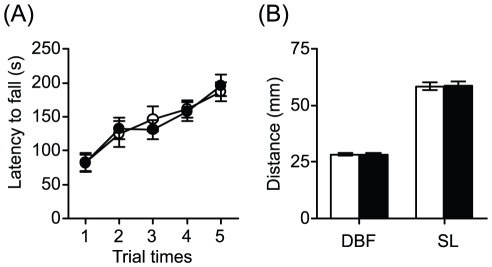
Motor performance in apelin-KO mice. (A) Rotarod test. The average for time spend on the rotarod across five test trials for wild-type (open circles, n = 22) and apelin-KO (closed circles, n = 22) mice. (B) Footprint analysis of wild-type (open column, n = 12) and apelin-KO (closed column, n = 14). The base of support was determined by measuring the distance between the central pads of the hind paws (DBF). The stride lengths of the hind paws (SL) were measured in two consecutive prints. Data are mean ± SEM.

**Figure 6:**
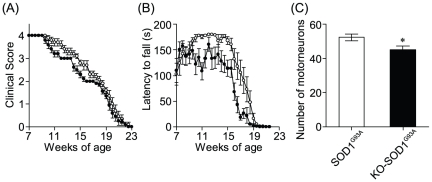
Effect of apelin deficiency on disease progression of ALS mice. (A) Motor signs (hind limb tremors) were measured as in Fig. 3B. (B) Rotarod performance of SOD1^G93A^ littermates (open circles) and KO-SOD1^G93A^ (closed circles) mice was measured as described in the methods for 180 s. (C) The number of motorneurons was decreased in the lumbar spinal cord of KO-SOD1 ^G93A^ mice at 14 weeks-old (n = 3). Data represent mean ± SEM. *p<0.05 vs. SOD1 ^G93A^ mice.

To evaluate the effects of apelin deficiency on the SOD1^G93A^ neurodegenerative phenotype, we compared double mutant apelin-KO and SOD1^G93A^ (KO-SOD1^G93A^) mice with their SOD1^G93A^ littermates in motor performance. We previously reported that apelin-KO mice exhibited normal behavioral phenotype other than ocular abnormality [16]. We also performed the rotarod and footprint tests recording locomotion which displayed comprised motor functions and gait disturbance of mice. Consistent with our previous data [16], we could not detect the abnormality of the motor performance in apelin-KO mice with intact SOD1 (Fig. 5). On the other hand, KO-SOD1^G93A^ mice displayed hindlimb tremors earlier than SOD1^G93A^ littermates (Fig. 6A). In the rotarod test, SOD1^G93A^ littermates had a learning phase showing improvement until 11 weeks of age, sustained their maximal performance level, and began to decline steadily at 14 weeks of age. In contrast to SOD1^G93A^ littermates, KO-SOD1^G93A^ mice had a learning phase until 9 weeks of age and subsequently performed significantly less well than SOD1^G93A^ littermates (Fig. 6B). Moreover, we investigated the number of motor neurons in lumbar spinal cord of KO-SOD1^G93A^ mice and SOD1^G93A^ littermates at 14 weeks of age by ChAT immunostaining. The number of ChAT-positive neurons in KO-SOD1^G93A^ mice was significantly decreased compared with SOD1^G93A^ littermates (Fig. 6C).

**Figure 7:**
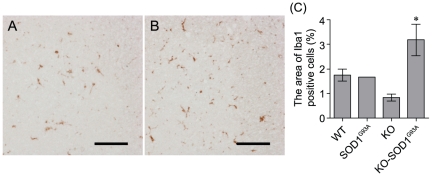
Microglial activation in apelin deficient mice with mutant SOD1. Representative pictures show that Iba1-positive microglia in the lumbar spinal cord of SOD1 ^G93A^ (A) and KO-SOD1^G93A^ (B) mice. (C) The Iba1-positive area was increased in the lumbar spinal cord of KO-SOD1^G93A^ mice at 14 weeks-old (n = 3). Scale bar = 50 µm. *p<0.05 vs. SOD1^G93A^. Data represent mean ± SEM.

Microglial activation contributes to the oxidative stress and damage involved in the ALS process, and parallels the disease progression [18]. Also, the expression of mutant SOD1 in microglia contributes to the progression motor neuron degeneration [19], [20]. In fact, we detected that the microglia of SOD1^G93A^ mice in lumber spinal cord were significantly activated at 18 weeks of age (WT: 2.64±0.22%, SOD1^G93A^: 6.75±2.16%). Therefore, to evaluate the disease progression, we next examined the microglia activation in lumbar spinal cord of KO-SOD1^G93A^ mice and SOD1^G93A^ littermates at 14 weeks of age. The Iba1^+^ microglial cell population in the lumbar spinal cord of KO-SOD1^G93A^ mice was significantly increased two-fold compared with SOD1^G93A^ littermates (Fig. 7). These data suggest that apelin deficiency promotes the disease progression of SOD1^G93A^ mice.

### Neuroprotective effect of apelin on cell death induced by hydrogen peroxide in primary neurons

**Figure 8:**
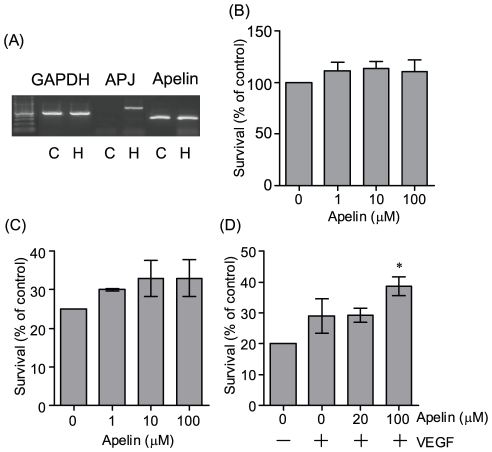
Effect of apelin and VEGF on oxidative stress-induced neurotoxicity in rat primary neurons. (A) Representative picture shows the expression of APJ and apelin mRNA in primary cortical and hippocampal neuronal cultures (representatively, C and H). Culture medium was replaced with neurobasal media without B27 supplement for 24 hours. (B) Cell viability of hippocampal neuronal cultures treated with indicated concentration of apelin for 24 hours. The cultures were treated with the indicated concentration of apelin (C) or with VEGF (50 ng/ml) and apelin (indicated concentration) (D) for 15 min prior to exposure to H_2_O_2_ (10 µM) for 24 hours. Cell viability was assessed by MTT assay (n = 4). *p<0.05 vs. control. Data represent mean ± SEM.

We next examined whether apelin protects neurons against cell death. Consistent with previous report [14], we detected the robustly expressed APJ in rat primary hippocampus neurons, not cerebral cortex neurons, by RT-PCR (Fig. 8A). The cultured hippocampal neurons were exposed to 10 µM hydrogen peroxide, which is known to induce apoptosis for 24 hours, and then cell death was measured 24 h later by 3- (4, 5-dimethylthiazol-2-yl)-2, 5-diphenyltetrazoilium bromide (MTT) assay. Apelin alone did not affect on cell viability in the absence of hydrogen peroxide (Fig. 8B). Moreover, Apelin alone (1–100 µM) did not protect hydrogen peroxide-induced cell death (Fig. 8C). However, 100 µM apelin co-applied with VEGF (50 ng/ml) showed significant neuroprotection (Fig. 8D).

## Discussion

SOD1 mutants such as G93A lead to the blood-spinal cord barrier breakdown and subsequent reduction in blood flow through lumbar spinal cord [6], [21]. In VEGF^δ/δ^ mice exhibiting ALS like phenotype, baseline neural blood flow was also lower than that of control mice [5]. Chronic hypoxia has been linked to motor neuronal death in ALS [1], [22]. In addition, apelin expression was upregulated by hypoxia in astrocyte (data not shown) as well as other cell types [12], [13]. Therefore, we predicted that apelin expression would be upregulated in the spinal cord of SOD1^G93A^ mice. Unexpectedly, we found that apelin expression in spinal cord was declined along with the progression of ALS. Mutant SOD1 including G93A disrupts the RNA stabilization, possibly by altering the ribonucleoprotein complex, and downregulates VEGF expression [23]. This dysregulation is mediated through the adenylate/uridylate-rich elements (AREs) in the VEGF 3′-untranslated region (UTR), where an aberrant ribonucleoprotein complex is formed in the presence of mutant SOD1. In *silico* analysis, we found that the apelin 3′-UTR also have five AREs (unpublished). Accordingly, the decline of apelin expression in spinal cord may be due to the disruption of RNA stabilization by mutant SOD1.

The APJ expression was not changed in spite of a decrease in ChAT neurons in the ALS mouse spinal cord, although the APJ expressed in ChAT-positive neurons in the spinal cord. APJ is expressed in not only ChAT-positive motor neurons but also interneurons in the gray matter of the spinal cord (Fig. 2). Therefore, the reason why we could not detect the decrease of APJ expression may result from APJ abundantly expressed in other neurons such as interneurons with the exception of motor neurons. On the other words, apelin/APJ system may have not only the neuroprotective effect, as suggested here, but also other roles such as the regulation of reflex or nerve conduction. In fact, we detected that the latency to lick the hind paw of apelin-KO mice was significantly decreased that of wild-type mice using 55°C hot plate test (wild-type: 26.3±1.31 s, apelin-KO: 22.1±1.56 s).

The distribution profiles were different from each other between mRNA expression of apelin and APJ in murine brains, although the highest expressions of apelin and APJ were observed in the spinal cord (Fig. 1). One possibility is that apelin could additionally act on receptors other than APJ in both the pons/medulla and the midbrain with enrichment of white matter. Alternatively, APJ could play a role other than apelin receptors in the cerebellum with a lower expression of apelin.

Apelin which activates the PI3K/AKT signaling pathway enhances angiogenesis induced by VEGF which also has a neuroprotective effect through VEGFR-2/AKT signaling pathway [16], [17]. Therefore, we examined whether apelin significantly enhances the neuroprotective effect of VEGF (Fig. 8). Apelin expectedly had a neuroprotective effect combined with VEGF, although further study on the relation between the apelin/APJ system and VEGF signaling is required. Meanwhile, a previous report also shows that a low dose of apelin (1–5 nM) significantly protects *quinolinic acid*-induced neuronal apoptosis in mouse cortical neurons [15]. In this study, we could not detect the neuroprotective effect of apelin alone (1–100 µM) against *hydrogen peroxide*-induced neuronal death in rat hippocampal neurons, and that was unexpected. The difference of neuroprotective effect could be explained on the basis of experimental conditions used such as treatment or species difference, or high doses of apelin (1 µM). This induces not only the activation of cell survival signaling such as ERK1/2 and AKT activation [24], [25], but also AMP-activated protein kinase (AMPK) activation [26]. Therefore, we cannot deny the possibility that the neuroprotective effect of apelin could be masked by activating other signaling pathways.

Currently, existing treatment for ALS provides only marginal benefit [27]. Insulin like growth factor-1 (IGF-1) is one of the most promising factors as neurotrophic factors against ALS-related neuronal death [28]. However, intramuscular- or intrathecal administration of IGF-1 had no significant effect on disease progression of SOD1^G93A^ mice [29], and subcutaneous IGF-1 has not shown benefit in 2-year ALS trial [30]. Furthermore, several factors, including lithium and BDNF, also delay the disease progression, but not completely protect the neuronal death in ALS [31], [32]. These results indicate that it is difficult for single factor to improve disease progression or survival of ALS. Our data that apelin enhanced the neuroprotective effect of VEGF on oxidative stress-induced neuronal death suggest a new cocktail therapy for ALS.

In conclusion, this is the first experimental study addressing the involvement of an endogenous apelin in the pathogenesis of ALS by using SOD1^G93A^ mouse model, familial ALS model. Oxidized species of wild-type SOD1 acquired 3′-UTR binding and toxic properties of ALS-linked mutant SOD1, implying that wild-type SOD1 may be a contributor of pathogenesis in sporadic ALS [33]. Although further studies are required to determine the mechanisms of apelin/APJ system in pathogenesis of ALS, impairment of posttranscriptional processing of apelin RNA by mutant or oxidized SOD1 may block an important neuroprotective pathway and accelerates motor neuron degeneration.

## Materials and Methods

### Animals

#### Ethics Statement

The animal experiments were performed in accordance with the guidelines of the Japanese Society for Pharmacology and were approved by the Committee for the Ethical Use of Experimental Animals at Setsunan University (approval ID: K08-13/08.04.14.2.S.017). All efforts were made to minimize animal suffering, reduce the number of animals used, and utilize alternatives to *in viv*o techniques.

C57BL/6 mice with targeted disruption of the apelin gene (apelin-KO) were generated as described previously [17]. Male mice expressing SOD1^G93A^ (Jackson Laboratory #002726) was backcrossed to C57BL/6 for at least 6 generations before the start of the experimentation with the apelin-KO mice. We crossbred male SOD1^G93A^ with female apelin^+/−^ to assess the effect of endogenous apelin deficient on motor performance in SOD1^G93A^ mice. Apelin gene is located in chromosome X. Hence, all analyses were performed with male littermates. Genotyping analysis was performed in accordance with the Jackson Laboratory protocols for SOD1^G93A^ mice and as previously described for apelin gene [17]. These mice were housed in metallic breeding cages in a room with a 12 h/12 h light/dark cycle. The humidity was 55%, temperature was 23°C, and mice had free access to food and water.

### Quantitative PCR to measure transcription levels

Mice were euthanized and lumbar spinal cords were removed. Total RNA extraction from lumbar spinal cord and reverse transcription of total RNAs (1 µg) were performed as described previously [34]. For gene expression analysis in cell culture, total RNA extraction from primary neurons, and reverse transcription of total RNAs (1 µg) were performed. Quantification of all gene transcripts was conducted using quantitative real-time RT-PCR with ABI Prism 7900-HT (Applied Biosystems; Foster City, CA). Real-time RT-PCR was performed using SYBR premix Ex Taq II (Takara, Ohtsu, Japan) with the use of primer pairs as previously described [34].

### Immunohistochemistry

Perfusion-fixed spinal cords were embedded in paraffin, and serial transverse sections (3 µm) through the lumbar spinal cord (L2–L4) were cut. Immunostaining for APJ, NeuN, GFAP, activated microglia (IbaI), and ChAT was performed using rabbit polyclonal anti-APJ as previously described [32], mouse monoclonal anti-neuronal nuclei (1∶300, Millipore), mouse monoclonal anti-GFAP (Progen), rabbit polyclonal anti-Iba1 (WAKO), and rabbit polyclonal anti-ChAT (1∶350, Millipore). Secondary antibodies were Alexa 568-conjugated anti-rabbit IgG (Molecular Probes), biotin-labeled anti-mouse IgG (1∶200, Dako) and FITC-conjugated streptavidin (1∶200, BD Bioscience), or peroxidase-conjugated anti-rabbit IgG (Nichirei) and Immpact DAB (Vector Laboratories). Immunostained sections were photographed using a fluorescence microscope (AZ-100M, Nikon, Japan). Preimmunized rabbit immunoglobulins were used as a negative control to confirm specific staining.

For the number of motor neurons, we counted the total number of the ChAT-positive cell bodies in 8 sections of lumber spinal cord (L2–4) selected every 100 µm. For quantification of microglial activation, we measured the percentage of the area of IbaI positive cells in the gray matter of lumber spinal cord (L2–L4).

### Behavioral test

Beginning at 7.5 weeks, all animals (n = 10/genotype) were weighed and evaluated for signs of motor deficit with the following 4 point scoring system twice a week (clinical score). 4 points if normal, 3 points if hind limb tremors are evident when suspended by the tail, 2 points if gait abnormalities are present, 1 point for dragging of at least one hind limb, 0 points for inability to right itself within 30 s.

For the rotarod test of evaluating the paralysis phenotype, the time for which an animal could remain on the rotating cylinder of a rotarod apparatus (Ugo Basile) at a constant speed of 12 rpm was measured. Each animal was given three tries and the longest latency to fall was recorded. 180 s was chosen as the arbitrary cut-off time. For phenotype analysis of the apelin-KO mice with intact SOD1, an accelerated rotarod was used. The apparatus has an initially speed of 2 rpm and gradually accelerated at a rate of 0.11 rpm/s. Each animal was given five trials; interval time was 30 min; 300 s was chosen as a arbitrary cut-off time.

The footprint test was used to qualitatively compare the gait of wild-type and apelin-KO mice. Footprint tests were performed at 8–12 weeks age of mice. To obtain footprints, the hind and forefeet of the mice were coated with black nontoxic paints. The animals were then allowed to walk along a 40-cm-long, 5-cm-wild runway coated with a white paper. Each mouse had three trials.

### Cell culture

Primary rat hippocampal cultures were prepared from embryonic day 17 Wister rat pups as previously described [35]. Cells were plated at a density of 5×10^5^ cells/ml on culture dish pre-coated with polyethylenimine and maintained in neurobasal media with B27 supplement (Invitrogen) at 37°C/5% CO_2_ air, as previously described [36]. After 8 days, the percentage of neurons was assessed by counting the Microtubule-associated protein-2 (MAP-2) positive neurons, and then we could detect more than 95% neurons in primary neuronal culture.

### Assessment of cell viability

Culture medium was replaced with neurobasal media without the B27 supplement for 24 h. The cultures were treated with apelin (Peptide institute Inc.) or with VEGF (Peprotech) and apelin for 15 min prior to exposure to H_2_O_2_ for 24 h. Cell viability was determined by the colorimetric MTT assay. After exposure to H_2_O_2_, 0.5 mg/ml MTT was added and incubation carried out for an additional 1 h. Then, 100 µl of dimethyl sulfoxide was added to dissolve the formazan particles. Finally, absorbance at 570 nm was measured with a microplate reader.

### Statistical analysis

Statistical analysis of the experimental data was performed by two-way analysis of variance followed by the Tukey–Kramer test (Fig. 3, 4, 5, 6), Student's *t* test (Fig. 6C), or one-way ANOVA followed by the Dunnett's test (Fig. 7, 8).

## References

[1] Cleveland DW, Rothstein JD (2001). From Charcot to Lou Gehrig: deciphering selective motor neuron death in ALS.. Nat Rev Neurosci.

[2] Rosen DR, Siddique T, Patterson D, Figlewicz DA, Sapp P (1993). Mutations in Cu/Zn superoxide dismutase gene are associated with familial amyotrophic lateral sclerosis.. Nature.

[3] Gurney ME, Pu H, Chiu AY, Dal Canto MC, Polchow CY (1994). Motor neuron degeneration in mice that express a human Cu,Zn superoxide dismutase mutation.. Science.

[4] Rothstein JD, Van Kammen M, Levey AI, Martin LJ, Kuncl RW (1995). Selective loss of glial glutamate transporter GLT-1 in amyotrophic lateral sclerosis.. Ann Neurol.

[5] Oosthuyse B, Moons L, Storkebaum E, Beck H, Nuyens D (2001). Deletion of the hypoxia-response element in the vascular endothelial growth factor promoter causes motor neuron degeneration.. Nat Genet.

[6] Zhong Z, Deane R, Ali Z, Parisi M, Shapovalov Y (2008). ALS-causing SOD1 mutants generate vascular changes prior to motor neuron degeneration.. Nat Neurosci.

[7] Wang Y, Mao XO, Xie L, Banwait S, Marti HH (2007). Vascular endothelial growth factor overexpression delays neurodegeneration and prolongs survival in amyotrophic lateral sclerosis mice.. J Neurosci.

[8] Iłzecka J (2004). Cerebrospinal fluid vascular endothelial growth factor in patients with amyotrophic lateral sclerosis.. Clin Neurol Neurosurg.

[9] O'Dowd BF, Heiber M, Chan A, Heng HH, Tsui LC (1993). A human gene that shows identity with the gene encoding the angiotensin receptor is located on chromosome11.. Gene.

[10] Tatemoto K, Hosoya M, Habata Y, Fujii R, Kakegawa T (1998). Isolation and characterization of a novel endogenous peptide ligand for the human APJ receptor.. Biochem Biophys Res Commun.

[11] Medhurst AD, Jennings CA, Robbins MJ, Davis RP, Ellis C (2003). Pharmacological and immunohistochemical characterization of the APJ receptor and its endogenous ligand apelin.. J Neurochem.

[12] Kunduzova O, Alet N, Delesque-Touchard N, Millet L, Castan-Laurell I (2008). Apelin/APJ signaling system: a potential link between adipose tissue and endothelial angiogenic processes.. FASEB J.

[13] Cox CM, D'Agostino SL, Miller MK, Heimark RL, Krieg PA (2006). Apelin, the ligand for the endothelial G-protein-coupled receptor, APJ, is a potent angiogenic factor required for normal vascular development of the frog embryo.. Dev Biol.

[14] O'Donnell LA, Agrawal A, Sabnekar P, Dichter MA, Lynch DR (2007). Apelin, an endogenous neuronal peptide, protects hippocampal neurons against excitotoxic injury.. J Neurochem.

[15] Zeng XJ, Yu SP, Zhang L, Wei L (2010). Neuroprotective effect of the endogenous neural peptide apelin in cultured mouse cortical neurons.. Exp Cell Res.

[16] Kasai A, Shintani N, Kato H, Matsuda S, Gomi F (2008). Retardation of retinal vascular development in apelin-deficient mice.. Arterioscler Thromb Vasc Biol.

[17] Kidoya H, Ueno M, Yamada Y, Mochizuki N, Nakata M (2008). Spatial and temporal role of the apelin/APJ system in the caliber size regulation of blood vessels during angiogenesis.. EMBO J.

[18] Hall ED, Oostveen JA, Gurney ME (1998). Relationship of migroglial and astrocytic activation to disease onset and progression in a transgenic model of familial ALS.. Glia.

[19] Beers DR, Henkel JS, Xiao Q, Zhao W, Wang J (2006). Wild-type microglia extend survival in PU.1 knockout mice with familial amyotrophic lateral sclerosis.. Proc Natl Acad Sci U S A.

[20] Boillee S, Yamanaka K, Lobsiger CS, Copeland NG, Jenkins NA (2006). Onset and progression in inherited ALS determined by motor neurons and microglia.. Science.

[21] Garbuzova-Davis S, Saporta S, Haller E, Kolomey I, Bennett SP (2007). Evidence of compromised blood-spinal cord barrier in early and late symptomatic SOD1 mice modeling ALS.. PLoS One.

[22] Ischiropoulos H, Beckman JS (2003). Oxidative stress and nitration in neurodegeneration: cause, effect, or association?. J Clin Invest.

[23] Lu L, Zheng L, Viera L, Suswam E, Li Y (2007). Mutant Cu/Zn-superoxide dismutase associated with amyotrophic lateral sclerosis destabilizes vascular endothelial growth factor mRNA and downregulates its expression.. J Neurosci.

[24] Masri B, Lahlou H, Mazarguil H, Knibiehler B, Audigier Y (2002). Apelin (65–77) activates extracellular signal-regulated kinases via a PTX-sensitive G protein.. Biochem Biophys Res Commun.

[25] Masri B, Morin N, Cornu M, Knibiehler B, Audigier Y (2004). Apelin (65–77) activates p70 S6 kinase and is mitogenic for umbilical endothelial cells.. FASEB J.

[26] Yue P, Jin H, Aillaud M, Deng AC, Azuma J (2010). Apelin is necessary for the maintenance of insulin sensitivity.. Am J Physiol Endocrinol Metab.

[27] Mitsumoto H (2007). A strategy to develop effective ALS therapy.. Brain Nerve.

[28] Kaspar BK, Lladó J, Sherkat N, Rothstein JD, Gage FH (2003). Retrograde viral delivery of IGF-1 prolongs survival in a mouse ALS model.. Science.

[29] Chian RJ, Li J, Ay I, Celia SA, Kashi BB (2009). IGF-1:tetanus toxin fragment C fusion protein improves delivery of IGF-1 to spinal cord but fails to prolong survival of ALS mice.. Brain Res.

[30] Sorenson EJ, Windbank AJ, Mandrekar JN, Bamlet WR, Appel SH (2008). Subcutaneous IGF-1 is not beneficial in 2-year ALS trial.. Neurology.

[31] Ishiyama T, Klinkosz B, Pioro EP, Mitsumoto H (1997). Genetic transfer of the wobbler gene to a C57BL/6J×NZB hybrid stock: natural history of the motor neuron disease and response to CNTF and BDNF cotreatment.. Exp Neurol.

[32] Fornai F, Longone P, Cafaro L, Kastsiuchenka O, Ferrucci M (2008). Lithium delays progression of amyotrophic lateral sclerosis.. Proc Natl Acad Sci U S A.

[33] Ezzi SA, Urushitani M, Julien JP (2007). Wild-type superoxide dismutase acquires binding and toxic properties of ALS-linked mutant forms through oxidation.. J Neurochem.

[34] Kasai A, Ishimaru Y, Kinjo T, Satooka T, Matsumoto N (2010). Apelin is a crucial factor for hypoxia-induced retinal angiogenesis.. Arterioscler Thromb Vasc Biol.

[35] O'Donnell LA, Agrawal A, Jordan-Sciutto KL, Dichter MA, Lynch DR (2006). Human immunodeficiency virus (HIV)-induced neurotoxicity: roles for the NMDA receptor subtypes.. J Neurosci.

[36] Yoshioka Y, Takeda N, Yamamuro A, Kasai A, Maeda S (2010). Nitric oxide inhibits lipopolysaccharide-induced inducible nitric oxide synthase expression and its own production through the cGMP signaling pathway in murine microglia BV-2 cells.. J Pharmacol Sci.

